# Comparison of the Personality Traits of Male and Female BASE Jumpers

**DOI:** 10.3389/fpsyg.2018.01665

**Published:** 2018-09-18

**Authors:** Erik Monasterio, Omer Mei-Dan, Anthony C. Hackney, Robert Cloninger

**Affiliations:** ^1^Canterbury District Health Board, Christchurch, New Zealand; ^2^Department of Orthopedics, University of Colorado Boulder, Boulder, CO, United States; ^3^Department of Nutrition, University of North Carolina at Chapel Hill, Chapel Hill, NC, United States; ^4^Department of Psychiatry, Washington University in St. Louis, St. Louis, MO, United States

**Keywords:** extreme sport, BASE jumping, sex differences, temperament, character

## Abstract

BASE jumping is an extreme adventure sport which consists of jumping from a fixed object with specially adapted parachutes. A few studies of the personality of BASE jumpers have been conducted, but little is known about how the women in this sport compare to the men. The purpose of this study is to compare the personality traits among a sample of men and women who are experienced BASE jumpers, as this provides an interesting and important opportunity to better understand the motivation for extreme sports. Eighty-three participants completed the Temperament and Character Inventory the day before the jump at the New River Gorge Bridge Day BASE Jumping event, West Virginia, United States. The sample included 64 men and 19 women. Results show that men and women BASE jumpers shared similar personality traits both in terms of temperament and character, except for the character trait of cooperativeness on which women scored higher than men. This suggests that the basic drive for participation in extreme sports is self-regulation of personal emotional drives and needs for self-actualization, rather than to oppose social pressure or cultural bias against female participation. These findings are discussed in relation with other studies conducted among extreme athletes and in terms of congruence between personality and activity.

## Introduction

BASE jumping is an extreme adventure sport which consists of jumping from a fixed object with specially adapted parachutes. “BASE" is an acronym for the four categories of fixed objects from which one can jump: Building, Antenna, Span (bridge, arch, or dome) and Earth (a cliff or other natural formation). This sport developed out of skydiving and is considered as one of the most dangerous extreme sports because of the risk of accident or death involved in the practice (Brymer, 2010; Mei-Dan et al., 2012). The injury rate estimates of 0.2–0.4% (Søreide et al., 2007; Monasterio and Mei-Dan, 2008; Mei-Dan et al., 2012) and the fatality risk estimates of 0.04% per jump (Søreide et al., 2007) and 1.7% per participant and per year (Westman and Björnstig, 2007). In addition, more than 70% of the participants report that they have witnessed the death or serious injury of another participant in the sport (Mei-Dan et al., 2012; Monasterio et al., 2012). Epidemiological data and injury reports are still scarce and may be underestimates (Mei-Dan et al., 2012; Søreide, 2012). BASE jumping is much more dangerous than skydiving (Søreide et al., 2007; Mei-Dan et al., 2012). It is practiced at a lower altitude than skydiving, which involves less aerodynamic control and more flight instability, and BASE jumpers jump with a single canopy with no reserve parachute (Monasterio and Mei-Dan, 2008; Mei-Dan et al., 2012). Because of the high risk associated with BASE jumping, it is prohibited in most public places. BASE jumping can be considered as a highly demanding activity in terms of training, discipline, and control (Brymer, 2010). It requires the participants to learn about the sport, about the characteristics of the site where they plan to jump, about the environmental conditions, and finally about themselves (e.g., Brymer, 2010). For example, participants must develop an in-depth understanding of equipment and its use, glide and flight techniques, and environmental parameters such as the strength and direction of the wind, pressure, altitude, natural formation, etc. In addition, there are many rules to apply while jumping, such as legal policies related to the specific site, and broader ethical rules (see more details on the official BASE jumping at http://www.cawp.rutgers.edu/). They also need to acquire knowledge about their own reaction under extreme conditions in order to make the right decisions and maintain safe behavior throughout the jump.

Extreme athletes have often been described as persons who tend to search for high risk experiences involving elevated levels of sensation, physiological arousal, and novelty (Zuckerman, 1983; Freixanet, 1991; Roberti, 2004; Monasterio et al., 2012, 2014). They have also been described as self-confident and optimistic individuals who tend to attribute accidents and fatalities to internal characteristics rather than to external circumstances (Morrongiellon and Rennie, 1998; Cloninger et al., 2006; Laurendeau, 2006, 2011). Even though, it has been argued that extreme athletes and BASE jumpers were not free from fear, in particular when close to the jump or risky event, it has been shown that they tend to underestimate risks and to show an optimism bias regarding their personal risk of being injured (Martha et al., 2010).

Accordingly, personality studies have demonstrated that most BASE jumpers were both high on the temperament traits of novelty and sensation seeking and low on harm avoidance. Temperament traits refer to automatic responses to emotional stimuli whereas character traits refer to voluntary goals and values. Novelty seeking is an automatic and biased response to novelty which is moderately heritable and which is reflected by impulsive decision making, active avoidance of frustration, extravagance in approach to cues of reward, and quick loss of temper. Whereas novelty seeking is a heritable bias in the activation of behaviors, harm avoidance is a heritable bias in the inhibition or cessation of behaviors and it is reflected through pessimistic worry, passive avoidance behaviors, shyness with strangers, and fatigability (Cloninger et al., 1993). Consequently, a combination of both high novelty seeking and low harm avoidance predicts impulsive, extraverted, and disinhibited behaviors (Cloninger, 2004). Accordingly, high-risk activity might be a way to regulate affects for those persons who score high on novelty seeking and who are not restrained by harm avoidance (Castanier et al., 2010a).

In addition, extreme athletes may also pursue higher-level motives, such as goal achievement, mastery-seeking, social motivation, connection with the natural environment, feeling of pleasurable bodily sensations, achievement of unselfconsciousness (Brymer, 2010; Buckley, 2012; Kerr and Mackenzie, 2012). As an example, Holmbom et al. (2017) conceived proximity flying as a transformative experience that changes one’s perspective on life, one’s skills and sense of identity. In terms of character, BASE jumpers often are highly self-directed and mature individuals who score highly on self-directedness and cooperativeness scales though they have extreme scores on temperament scales (Monasterio et al., 2012, 2016). Put another way, strong character traits allow the self-regulation of extreme temperament traits, which is crucial to minimize the risks of BASE jumping. Such findings are interesting because it has been shown that even if temperament (i.e., measured by novelty seeking, harm avoidance, reward dependence, and persistence) constrains character development (i.e., measured by self-directedness, cooperativeness, and self-transcendence) (Cloninger et al., 1997), character development is more predictive of health and well-being than temperament (Cloninger et al., 1997; Josefsson et al., 2011). Hence, people with similar temperament profiles, which correspond to emotional drives and automatic behavioral responses, can behave in distinct ways and can be either healthy, happy, and well-integrated in society; or with poor health, psychopathological features, and maladaptation to the society, depending on the development of their character (Cloninger and Zohar, 2010). Accordingly, the conscious goal BASE jumpers pursue by acting in a certain way will be reflected by their character development rather than by their temperament profile, even though their behaviors may also be driven by impulses and emotions which are reflected by high scores on temperament traits.

However, studies conducted among BASE jumpers and other extreme sports usually don’t consider gender differences and little is known about female participants of extreme sport. As a matter of fact, women are even sometimes excluded from the studies on high-risk sports because of their low frequency (Castanier et al., 2010a,b; Martha et al., 2010). 90% of BASE jumpers are actually males (Søreide et al., 2007), and men represent about 81 to 92% of cases of injuries and fatalities (Westman and Björnstig, 2007; Mei-Dan et al., 2012; Søreide, 2012; Mei-Dan et al., 2013). Published results usually aggregate subjects regardless of gender, so it is uncertain how similar the minority of women are to the men in these samples.

The differences of prevalence between men and women in extreme sports are not really surprising. On the one hand, women have for a long time been less involved than men in sport and extreme sports because of culture and gender roles (Lopiano, 2000; Gieseler, 2012). On the other hand, studies on personality in samples representative of the general population have found that women tend to score slightly lower than men on the temperament dimensions of novelty seeking or sensation seeking and slightly higher than men on harm avoidance. They also score substantially higher on reward dependence and cooperativeness, and slightly higher in self-transcendence traits (Cloninger et al., 1993; Feingold, 1994; Miettunen et al., 2007; Fresán et al., 2011; Del Giudice et al., 2012; Cross et al., 2013; Josefsson et al., 2013).

The gender difference in the prevalence of BASE jumping provides an interesting and important opportunity to better understand the motivation for extreme sports, as has been considered for personality disorders and alcohol use disorders in other work (Cloninger et al., 1978). What is the basic motive that drives men and/or women to take the risks involved in extreme sports despite cultural obstacles? If the motivation is to oppose cultural norms, women would need to differ more extremely than men because the cultural opposition is greater to the participation of women, as is the case for gender differences in antisocial personality disorder (Cloninger et al., 1978). On the other hand, if the motivation is self-regulation of personal drives and needs for self-actualization, the genders would not be expected to differ because the strength or weakness of the predisposition for self-regulation has an internal locus of control unrelated to cultural opposition, as is the case for gender differences in alcohol use disorders (Cloninger et al., 1978).

Gender differences is a field of widespread interest in which the differences have been viewed from several perspectives, including evolution (Davies and Shackelford, 2006), biology, and sociology or culture (see for example Lyng, 1990; Feingold, 1994). As a matter of fact, while briefly retracing the history of risk-taking activity and extreme sports, Søreide (2012) found that such activity was used as a test of male courage and as a passage into manhood. In the same range of ideas, Lyng defines voluntary risk-taking in terms of negotiating with the “edge” which is the boundary between life and death, consciousness and unconsciousness, or the idea of testing the limits of body and mind (1990). He also describes edgework experience in terms of a search for self-determination, self-actualization and self-realization, which comes along with a specific sequence of emotions, and with an alteration in perceptions and consciousness (1990). In turn, risk-taking activity and sport in general had been considered as harmful for women’s health and for their reproductive health in particular (Lopiano, 2000; United Nation’s report on women, gender, equality and sport, 2007).

From an evolutionary perspective, differences between men and women have been explained in terms of sexuality and natural selection (Davies and Shackelford, 2006; Del Giudice et al., 2012). For instance, risk taking has been considered as a product of differential adaptive challenges among men and women (Davies and Shackelford, 2006). Such a view is argued to be complementary with the biosocial theory which addresses sex differences in terms of biological differences (e.g., anatomical attributes, hormones, rhythms, or cycles) and of contextual factors (i.e., social, economic, technological, and ecological forces) (Davies and Shackelford, 2006). According to the sociological perspective, gender differences result from societal norms regarding social roles and from the conformity with other’s expectancies which are often driven by stereotypical beliefs (i.e., social role model and expectancy model). Differences observed are also sometimes reduced to an artifact in the measurement, due to social desirability (Feingold, 1994).

Although there may have been different pressures shaping the personalities and biology of men and women over evolutionary time, the roles of women and men in society are much more similar now. As a result, women now have the same access to extreme sports that men do. Some laws such as the federal antidiscrimination law (title IX in 1972 in the United States) and the Amateur Sports Act of 1978 have contributed to open access to women in sport. Those laws came along with the recognition of sport benefits in terms of physical, mental, social, and spiritual health of women (Lopiano, 2000). For example, sport practice has been associated with an increase in confidence, self-esteem, academic success and a decrease in the initiation of high-risk health behaviors among women (Lopiano, 2000 for a brief review). As a consequence, women’s participation in sport has increased within the last few decades. For example, 17.8% of high school athletes were women in 1972 in the United States whereas they were 40% in 1996 (Lopiano, 2000). Similar trends have been observed among college and Olympics’ athletes (Lopiano, 2000). It is thus possible that the sociological context have allowed the expression of women’s drives and interest through sport. In a study conducted among male and female athletes (hockey players and figure skaters), Wiley and colleagues found for example that men and women had similar levels of overall involvement in sport. Women had a higher level of attraction to sport than men did, whereas men considered sport as more central in their life than women did (Wiley et al., 2000). In addition, Hardin and Greer (2009) found that action sports were considered both by men and women as a masculine sport, but also as a category of sport apart from what they named “hyper-masculine sports” (i.e., football, weightlifting, rugby, basketball), “neutral sports” (i.e., soccer, swimming, tennis), and “feminine sports” (volleyball, gymnastics). In fact, extreme sports such as BASE jumping don’t imply body contact and don’t require important physical strengths which are usually associated with masculine sports (Metheny, 1967; Wiley et al., 2000). Finally, even if women tend to score lower than men on novelty seeking and higher on harm avoidance on average, all kinds of personality profiles exists among both women and men. Thus, people sharing the same traits or personality profiles, regardless of their gender, are more likely to share similar drives, cognitions and to choose similar activities. In fact, previous studies have shown that people tend to choose activities that are congruent with their personality (Courneya and Hellsten, 1998; Sievert et al., 2016). Personality also predicts the individual motives to exercising (Ingledew and Markland, 2008) and has been shown to be a better predictor of motivations to exercise than gender was (Davis et al., 1995). It would not be surprising then to find that women have similar personality profiles than men among BASE jumpers.

Still, little is known about female BASE jumpers or other populations of extreme sportswomen. Interestingly, available studies have shown that women who are experienced in extreme sports may be similar to the men (Cazenave et al., 2007; Modroño and Guillen, 2011). For example, in their study among 79 high-level windsurfers, Modroño and Guillen (2011) found that women scored similarly to men on anxiety and self-confidence scales. Modroño and Guillén (2016) observed again that men and women windsurfers didn’t differ in terms of sport motivation, goal orientation, or physical self-concept. Cazenave et al. (2007) studied the purpose of the risky activity and found that women who were engaged in risk-taking sports for leisure purpose had a more “masculine” profile than women who were engaged in high-risk activities for professional purpose. Masculinity was here defined as a high level of leadership, sportsmanship, self-confidence, and a low level of consideration for others and tenderness (Bem, 1974). In other words, women whose profession involved risk-taking were described as having less masculine traits and more feminine ones in comparison to women engaged in high-risk activities for leisure purpose. Women who practiced their activity for a leisure purpose were also more impulsive and sensation seekers in comparison to women pursuing a professional purpose. Women at leisure more often used an escape from awareness strategy to regulate distress (i.e., turning attention away from the self or engaging in actions that reduce the level of self-awareness) and they used less often a compensation strategy (i.e., shifting emphasis from less rewarding self-definitions to more rewarding ones). This subgroup finally included more alexithymic individuals than the other one.

However, very few studies have investigated such gender differences among extreme athletes using a comprehensive model of personality, and, to our knowledge, no study comparing men and women have been conducted among BASE jumpers. The purpose of the study is to compare men and women who are experienced BASE jumpers using the Temperament and Character Inventory (TCI), a comprehensive model of personality.

### Temperament and Character Inventory (TCI)

The TCI is an inventory for personality traits for Cloninger’s psychobiological model of personality (Cloninger et al., 1993), which explains personality in terms of complex adaptive systems interacting with each other. Personality has been defined as “the dynamic organization within an individual of the psychobiological systems that modulate adaptation to a changing environment” (Cloninger et al., 1993) and also as “the way that people learn from experience and adapt their feelings, thoughts, and actions” (Cloninger et al., 1997). Such definition of personality includes systems regulating cognition, emotion, personal impulse control and social relations (Cloninger et al., 1997), and has been found to be the result of both heredity (i.e., genetics) and social environment and education (Cloninger et al., 1993). More precisely, the temperament has been defined in terms of automatic, preconceptual responses that are partially heritable and stable throughout life, and consists of four dimensions, namely, novelty seeking, harm avoidance, reward dependence, and persistence. Those temperament traits explain how persons respond to novelty, danger or punishment, and reward. *Novelty seeking* is viewed as a heritable bias in the activation or initiation of behaviors in response to novelty (e.g., impulsive decision making, exploratory activity); *Harm avoidance* is viewed as a heritable bias in the inhibition or cessation of behaviors (e.g., pessimistic worry, fear of uncertainty); *Reward dependence* is viewed as a heritable bias in the maintenance or continuation of ongoing behaviors (e.g., social attachment, dependence on approval of others); and *Persistence* corresponds to perseverance despite frustration and fatigue.

In contrast to temperament, character involves the conceptual organization of perceptions which influences behavioral goals and expectancies. Character expression is determined by our concepts of our identity as an autonomous individual, as a part of humanity and society, and as a part of the universe as a whole. One character dimension is *Self-directedness* which refers to the ability of an individual to control, regulate and adapt behavior to fit the situation in accordance with individually chosen goals and values, and which encompasses traits such as purposefulness, responsibility. *Cooperativeness* refers to the identification with and acceptance of other people and is related to agreeability, empathy, helpfulness to others without selfish domination. *Self-transcendence* is viewed as the identification with everything conceived as essential and consequential parts of a unified whole. It corresponds to a unitive perspective or consciousness where the person is simply aware of being part of a greater whole, and it can be described as acceptance, identification, or spiritual union with nature and its source (Cloninger et al., 1993, 1997).

In two previous studies among BASE jumpers, Monasterio et al. (2012, 2016) have studied personality using the TCI. In a first study among BASE jumpers in Monasterio et al. (2012) found that BASE jumpers scored higher than a control group on the temperament dimension of novelty seeking and lower on harm avoidance, which is consistent with results obtained in studies on sensation seeking and with studies emphasizing fear management. They also scored lower on reward dependence, which is a measure of social attachment and sentimentality. Furthermore, BASE jumpers scored higher on the character dimension of self-directedness, which is in line with previous discussions on goal achievement and self-realization. They finally scored lower on self-transcendence than the control group, which might be explained by the strong need for self-control involved in the practice. More recently, Monasterio et al. (2016) replicated the earlier results among a sample of BASE jumpers recruited at the New River Gorge Bridge Day BASE jumping event (except for the dimension of cooperativeness, which was here higher among BASE jumpers than in the control group) and identified several main multidimensional profiles in terms of temperament, character, and plasticity (for further details, see Monasterio et al., 2016). Taken together, those results suggest that not all BASE jumpers share the same personality profile or are driven by the same goals, even though most of the participants appear to share some common traits such as high novelty seeking, low harm avoidance, high self-directedness, and high cooperativeness.

This study assessed the personality of women BASE jumpers and compared this to their male counter parts.

## Materials and Methods

### Participants

One-hundred and sixty-two participants from different countries at two distinct Bridge Day events volunteered to participate in the study. All participants gave a written informed consent before completing the questionnaires. Data were collected from the 2008 (*n* = 62) and the 2014 (*n* = 100) Bridge Day events. Unique identifying codes, based on name initials and dates of birth were utilized to ensure participant data was not collected twice in 2008 and 2014. The overall number of participants at each event was around 400 participants. Participants were excluded from the study because of missing data on the TCI and 24 participants because of missing data on previous experience in BASE jumping (i.e., number of jumps and experience in years). Participants who had done less than 10 jumps prior to the event were excluded from the study (*n* = 46) in order to consider only regular BASE jumpers. The final sample was comprised of 83 participants, including 64 men (77%) and 19 women (23%) (see **Table 1**). The participants were between 20 and 79 years old (*M* = 36.78, *SD* = 11.91) and women were slightly older than men (*M* = 38.53, *SD* = 11.6 and *M* = 36.27, *SD* = 12.04, respectively). Most of the participants came from the United States and some others came from United Kingdom, Canada, Israel, and South Africa. In average, participants had been practicing BASE jumping for 6.7 years (6.1 [*SD* = 5.5] for men and 8.2 [*SD* = 7.7] for women) and the average number of jumps among participants was 132 (*SD* = 302.7). Men had experienced many more jumps than women in general (*M* = 159.0 [*SD* = 340] for men and *M* = 43.5 [*SD* = 43] for women).

**Table 1:** Demographics.

	Men	Women	Total
***N***	64	19	83
**Age**			
Mean	36.3	38.5	36.8
Standard deviation	12.0	11.6	11.9
Minimum	20	25	20
Maximum	79	64	79
**Time BASE jumping (years)**			
Mean	6.1	8.2	6.7
Standard deviation	5.5	7.7	6.1
Minimum	0.1	1	0.1
Maximum	20	28	28
**Total No. of jumps**			
Mean	159.0	43.5	132.5
Standard deviation	340.0	43.2	302.7
Minimum	10	10	10
Maximum	2300	200	2300

### Material

Participants provided demographical information and BASE jump information. They reported their number of BASE jumps per year and how long they had been involved in the sport.

They also completed the TCI (Cloninger et al., 1993). The TCI is composed of 240 true-false items that allows for the assessment of seven dimensions of personality and has already demonstrated strong internal consistency and test–rest reliability of its scales (*r* = 0.8 to 0.9) (Cloninger et al., 1994; Grucza and Goldberg, 2007).

### Procedure

Data for the study was collected from the New River Gorge Bridge Day BASE Jumping event, both in 2008 and 2014. This is an annual event in October in Fayetteville, WV, United States where participants jump from a bridge 876 feet above the New River. The researchers discussed the nature and purpose of the study at this event and invited voluntary participation from attendees. Participants completed several paper questionnaires the day before the jump without remuneration, as described in prior work (Monasterio et al., 2012). The participants and conditions at the two events were comparable with no suggestion of heterogeneity. IRB approval was obtained prior to commencement of each study (Monasterio et al., 2012, 2016).

### Statistical Analysis

Statistical analyses were carried out using SPSS for Windows, version 22.0. The Wilcoxon Mann–Whitney test was used as a non-parametric test for independent samples to compare the two samples of men and women because samples were small when divided by gender, and scores were not normally distributed.

## Results

Data collected in 2008 and in 2014 were combined in all analyses.

### Mean Values

BASE jumpers in this study scored above average on Novelty Seeking, Self-Directedness and Cooperativeness, and below average on Harm Avoidance, Reward Dependence and Self-Transcendence (**Table 2**).

**Table 2:** Distribution of the scores on the TCI scales among for all the participants (*n* = 83).

	NS	HA	RD	P	SD	C	ST
Mean	22.57	8.5	13.9	5.9	33.8	34.8	14.2
Median	22.00	7.00	15.00	6.00	35.00	36.00	13.00
Mode	18.0	4.0	15.0	5.0	40.0	40.0	20.0
Standard deviation	5.9	6.2	4.2	1.4	6.9	5.7	7.2
Variance	35.3	39.1	17.5	2.1	47.8	32.6	51.3
Minimum	12.0	.0	3.0	2.0	8.0	11.0	2.0
Maximum	35.0	26.0	23.0	8.0	43.0	41.0	31.0

*NS, novelty seeking; HA, harm avoidance; RD, reward dependence; P, persistence; SD, self-directedness; C, cooperativeness; ST, self-transcendence.*

### Gender Differences

Means and standard deviations of the seven temperament and character dimensions of the TCI for both men and women are shown in **Tables 3**, **4**.

**Table 3:** Descriptive data of men and women scores on the TCI.

	NS	HA	RD	P	SD	C	ST
**Mean**							
Men	22.7	8.1	13.7	6.0	33.7	34.1	13.6
Women	22.2	9.8	14.9	5.7	34.2	37.3	16.1
**Standard deviation**							
Men	6.0	6.4	4.4	1.4	7.2	6.2	6.8
Women	5.8	5.6	3.3	1.6	6.0	2.4	8.2
**Minimum**							
Men	12.0	0	3.0	2.0	8.0	11.0	2.0
Women	15.0	2.0	8.0	3.0	24.0	32.0	2.0
**Maximum**							
Men	35.0	26.0	23.0	8.0	43.0	41.0	29.0
Women	35.0	22.0	20.0	8.0	43.0	41.0	31.0

*NS, novelty seeking; HA, harm avoidance; RD, reward dependence; P, persistence; SD, self-directedness; C, cooperativeness; ST, self-transcendence.*

**Table 4:** Independent sample tests on the TCI, depending on sex.

	NS	HA	RD	P	SD	C	ST	Jumps	Years
Mann–Whitney *U*	569	476.5	512.0	550.5	601	426.5	505	413.5	499.5
Wilcoxon W	759	2556.5	2592	740.5	791	2506.5	2585	603.5	2579.5
*Z*	−0.42	−1.43	−1.0	−0.64	−0.08	−**1.97^∗^**	−1.12	−**2.11^∗^**	−1.18

*(a) Grouping Variable: sex. NS, novelty seeking; HA, harm avoidance; RD, reward dependence; P, persistence; SD, self-directedness; C, cooperativeness; ST, self-transcendence. ^∗^p < 0.05.*

**FIGURE 1:**
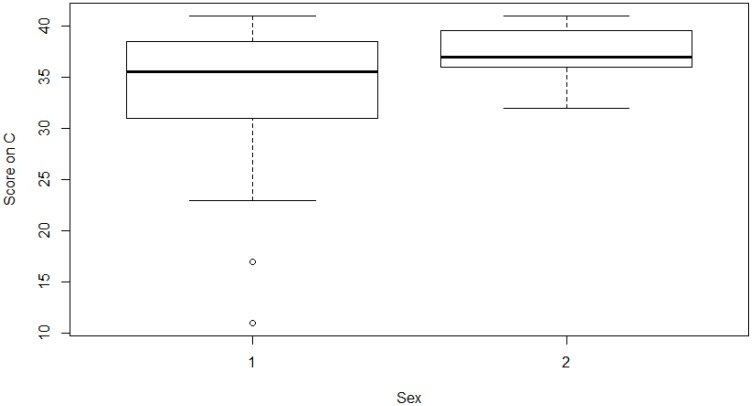
Box plots of the distribution of men and women BASE jumpers’ scores on cooperativeness. In each box, the central line is the median, the edges of the box are the 25^th^ and the 75^th^ percentiles, and the whiskers extend to the most extreme data points. All data was included in the analyses. 1 = Men; 2 = Women.

Results showed that men and women had similar scores on all of the TCI dimensions, except for the trait of cooperativeness where women scored higher than men (*z* = 1.97, *p* < 0.05; **Figure 1**).

## Discussion

The purpose of this study was to assess the personality of women BASE jumpers and identify possible differences in temperament and character traits when compared to their more prevalent male counterparts. We hypothesized that women would need to differ more extremely than men if their motivation is to oppose cultural biases for women to participate in extreme sports. Alternatively, we hypothesized that the genders would not differ if their motivation is satisfaction of personal drives and needs for self-actualization. Our results show that female BASE jumpers differ little in personality from male BASE jumpers, so we conclude that the basic drive for participation in extreme sports is self-actualization of personal emotional drives and needs, rather than cultural opposition. In fact, the only significant difference found in this study was on the trait of cooperativeness which is defined as being tolerant with others, agreeable, empathetic, helpful, and compassionate. Other studies have consistently shown that women in the general population score higher than men on the dimension of cooperativeness (e.g., Cloninger et al., 1993; Feingold, 1994; Fresán et al., 2011; Josefsson et al., 2013), and we find that female BASE jumpers also retain this difference from their male counterparts.

Without prospective follow-up of subjects as they continue to participate in BASE jumping, we cannot say with certainty whether some subgroups of participants change in personality as they increase in experience and self-confidence. However, we previously found that personality was associated directly with stress reactivity and with jumping history, but jumping history did not influence stress reactivity once personality was taken into account (Monasterio et al., 2016). The current findings suggest that prior personality (particularly, internal motivation for self-regulation) is the basic motivation to participate in BASE jumping, not an effort to oppose external opposition.

Our results are in line with other studies of gender differences in extreme sports. For example, in their study among windsurfers, Modroño and Guillen (2011) found that men and women were not different on their level of anxiety and self-confidence. In 2016 they found that this population had a high level of self-determined motivation, a positive physical self-concept, a strong task motivational orientation and a weak ego goal orientation. Again, no significant gender differences emerged (Modroño and Guillén, 2016).

What characterizes “femininity” has been defined and measured in term of consideration for others and tenderness while “masculinity” has been defined as a high level of leadership, sportsmanship, self-confidence, and a low level of consideration for others and tenderness (Bem, 1974; Holt and Ellis, 1998; Prentice and Carranza, 2002). However, women also generally score substantially higher on reward dependence and slightly higher than men on harm avoidance and self-transcendence (Cloninger et al., 1993; Feingold, 1994; Miettunen et al., 2007; Fresán et al., 2011; Del Giudice et al., 2012; Cross et al., 2013; Josefsson et al., 2013). Such differences are not observed in our sample of BASE jumpers, except for the trait of cooperativeness.

In addition, the degree of assertiveness or self-confidence, which is sometimes considered as masculine (Bem, 1974; Holt and Ellis, 1998; Prentice and Carranza, 2002), is not differentially expressed among men and women in our study where the level of self-directedness is similar among both sexes. Self-directedness has been defined as the ability of an individual to control, regulate, and adapt behavior to fit the situation in accordance with individually chosen goals and values (Cloninger et al., 1993). In fact, this trait has been shown to be among the strongest predictor of well-being and ill-being within the personality, along with cooperativeness and self-transcendence (Cloninger et al., 2010; Josefsson et al., 2011) regardless of gender. In addition, previous studies didn’t find any significant differences between men and women on self-directedness scores (Cloninger et al., 1993; Fresán et al., 2011; Josefsson et al., 2013). In other words, it seems that both men and women have this potential for self-directedness and cooperation, which are particularly elevated in the population of BASE jumpers (Monasterio et al., 2016). Both male and female BASE jumpers are usually higher in Novelty Seeking and lower in Harm Avoidance than the average of people in the general population. Considered together, personality characteristics of women and men BASE jumpers fit with the characteristics of the activity, the skills it requires, and also the kind of experience it offers. Extreme activity such as BASE jumping requires a high level of organization and self-directedness, as well as high self-control capacities along with a low level of fear when close to jump. This finding finally sustains other studies showing that men and women both tend to choose activities that are congruent with their personality (Courneya and Hellsten, 1998; Sievert et al., 2016). However, the fact that men and women BASE jumpers share common personality traits doesn’t mean that they are all alike. A previous study conducted among BASE jumpers has shown that there were several personality profiles among BASE jumpers, which are defined by the interaction between several personality traits (Monasterio et al., 2016). Further studies could explore the extent to which those personality profiles are similarly distributed or not among men and women.

### Application of the Findings

BASE jumping is a high risk sport with elevated rates of injuries and death (Søreide et al., 2007; Monasterio and Mei-Dan, 2008; Mei-Dan et al., 2012). The results of this study provide information on the population of women BASE jumpers, and thus may serve in the implementation of prevention and treatment plans. Understanding biases to learning and decision making based on the findings of personality traits can guide in developing adequate mental preparation for participation in high-risk activities. For example, findings of low Harm Avoidance particularly in combination with high Novelty Seeking suggests that BASE jumpers have a tendency to be optimistic and energetic in situations of danger and uncertainty, which may lead to excessive optimism in marginal conditions. Awareness of this bias and its effect on decision making at an individual level may influence toward more conservative approaches in these conditions, which may decrease the risk for adverse outcomes. It also contributes to the understanding of BASE jumpers and extreme athletes and to the body of literature on congruence between personality and activity choice.

### Limitations

The present study has some limitations. First, even combining data from two BASE jumping events, the sample size of regular female participants remains small. Only 19 women participants have been included in the study. The fact that the study included more men than women is quite representative of the repartition of men and women in BASE jumping, however 19 women participants remain a small sample size. Second, the population was not a random sample and only participants who volunteered to participate in the study completed the questionnaires. Finally, we chose to study only personality traits of the participants in this first study of sex differences among BASE jumpers. However, it has already been demonstrated that personality is a complex adaptive system which contains dimensions interacting with each other. In other words, personality can’t be reduced to the sum of individual traits but is rather defined as the expression of complex interactions between those traits, which is best measured as a multidimensional profile. Previous studies have found that not all BASE jumpers shared the same personality profile despite common traits (Monasterio et al., 2012, 2016). Further studies could investigate sex differences among a larger sample of BASE jumpers. A larger sample of participants would allow for the consideration of personality profiles in addition to personality traits, as it has been done in other works (Giakoumaki et al., 2016; Monasterio et al., 2016).

## Conclusion

This study is the first study assessing sex differences among BASE jumpers using a comprehensive model of personality. Results showed that within this population, women and men shared similar personality traits including both temperament and character, except for the character trait of cooperativeness in which women scored higher than men. At least among experienced BASE jumpers, men and women do not differ much in personality except that female BASE jumpers retain the general advantage of women being more cooperative than men. Such results indicate that the demands of BASE jumping result in comparable personality traits regardless of gender. Regardless of gender, BASE jumpers must have what Tom Wolfe called the “Right Stuff” (Monasterio et al., 2016) – willingness to take risks combined with the responsibility to discipline one’s self to minimize those risks. Nonetheless, further research on larger sample of female extreme athletes would allow further exploration of the heterogeneity of personality profiles within both male and female extreme athletes.

## Ethics Statement

This study was carried out in accordance with the recommendations of the following guidelines (IRB# 14-1942; approved 9/4/2014). Institutional Review Board approval was obtained prior to commencement of the study from the University of North Carolina at Chapel Hill Written consent was obtained from those who agreed to participate in the survey in accordance with the stipulated IRB procedure and in accordance with the Declaration of Helsinki.

## Author Contributions

EM and RC contributed to analysis and interpretation of data. AH, EM, and OM-D to data collection. All the authors to review and approval of the final manuscript.

## Conflict of Interest Statement

The authors declare that the research was conducted in the absence of any commercial or financial relationships that could be construed as a potential conflict of interest.
